# Attention Based CNN-ConvLSTM for Pedestrian Attribute Recognition

**DOI:** 10.3390/s20030811

**Published:** 2020-02-03

**Authors:** Yang Li, Huahu Xu, Minjie Bian, Junsheng Xiao

**Affiliations:** 1 School of Computer Engineering and Science, Shanghai University, Shanghai 200444, China; huahuxu@163.com (H.X.); xpceo@126.com (J.X.); 2 School of Information Technology, Shanghai Jianqiao University, Shanghai 201306, China; 3 Information Office, Shanghai University, Shanghai 200444, China; bianmj0302@aliyun.com

**Keywords:** pedestrian attribute recognition, convolutional neutral networks (CNN), conventional long short-term memory (ConvLSTM), channel attention (CAtt), multi-label classification

## Abstract

As a result of its important role in video surveillance, pedestrian attribute recognition has become an attractive facet of computer vision research. Because of the changes in viewpoints, illumination, resolution and occlusion, the task is very challenging. In order to resolve the issue of unsatisfactory performance of existing pedestrian attribute recognition methods resulting from ignoring the correlation between pedestrian attributes and spatial information, in this paper, the task is regarded as a spatiotemporal, sequential, multi-label image classification problem. An attention-based neural network consisting of convolutional neural networks (CNN), channel attention (CAtt) and convolutional long short-term memory (ConvLSTM) is proposed (CNN-CAtt-ConvLSTM). Firstly, the salient and correlated visual features of pedestrian attributes are extracted by pre-trained CNN and CAtt. Then, ConvLSTM is used to further extract spatial information and correlations from pedestrian attributes. Finally, pedestrian attributes are predicted with optimized sequences based on attribute image area size and importance. Extensive experiments are carried out on two common pedestrian attribute datasets, PEdesTrian Attribute (PETA) dataset and Richly Annotated Pedestrian (RAP) dataset, and higher performance than other state-of-the-art (SOTA) methods is achieved, which proves the superiority and validity of our method.

## 1. Introduction

Recently, along with the fast development of video surveillance networks, using computer technology to realize the intelligence of video surveillance systems has become a hot research area. As pedestrians are one of the important targets of video surveillance, the recognition of pedestrian visual attributes (such as gender, age and clothing style, etc.) has become an important task [1].

As an intermediate semantic feature, pedestrian visual attributes are robust to viewpoint changes and observation conditions. They can establish the relationship between low-level visual features and high-level cognition, and assist in many visual tasks such as human face recognition [2], person re-identification [3,4], person retrieval [5,6] and human identification [7]. Under real surveillance scenarios, pedestrian attribute recognition is a great challenge, and the main difficulties are: (1) poor image quality, low resolution, occlusion, motion blur, etc.; (2) some pedestrian attribute recognition tasks require local fine-grained information, such as “glasses”; (3) attribute appearance and spatial location are prone to change, such as different types of “bag”; (4) lack of large-scale datasets and unbalanced sample distribution.

In recent years, deep learning has made great progress in the field of computer vision, and the methods of pedestrian attribute recognition based on deep learning [8,9] have attracted the attention of researchers. Mainstream methods generally build models based on three factors: (1) exploring the correlation between attributes [10,11], such as “skirt” and “female” which have strong correlations, as this relationship can provide constraint reasoning for visual attributes; (2) using the attention mechanism [12,13] when predicting different attributes, enhancing the attention to the corresponding position or feature of the attribute (i.e., when predicting the attribute “glasses”, enhance the attention to the head or face); (3) exploring visual context information [14,15] as an additional auxiliary information source for pedestrian attribute recognition, since different people have a higher probability of having the same kind of attributes in the same scene (i.e., most people wear sportswear on the playground). However, the existing methods of pedestrian attribute recognition are insufficient to explore the correlation and spatial relationship between attributes, and they ignore the differences in the amount of information contained in attributes, resulting in the unsatisfactory performance of current pedestrian attribute recognition methods.

Aiming to address the problems mentioned above, this paper considers pedestrian attribute recognition as a task of spatiotemporal sequence and multi-label image classification, and presents a novel convolutional neural network (CNN), channel attention (CAtt) and convolutional long short-term memory (ConvLSTM) model (CNN-CAtt-ConvLSTM). Specifically, the model is composed of a CNN for attribute feature extraction, a CAtt mechanism for re-adjusting the correlated feature responses of pedestrian attributes, and ConvLSTM for mining correlation and spatial information between different attributes. The main contributions and novelty of this paper are as follows:

- Existing methods use Recurrent Neural Network (RNN) or LSTM to excavate the correlation of pedestrian attributes. The spatial information of attributes is lost during this procedure, however, spatial information is important to improve the performance of pedestrian attribute recognition. In this paper, in order to better mine the spatial and semantic correlation between attributes, ConvLSTM, which can retain the spatial information by using convolution operation in input-to-state and state-to-state transition, is adopted. An end-to-end trainable model is established by superposing several ConvLSTMs to extract spatiotemporal correlation information from the predicted pedestrian attribute sequence.
- CNN is used as a visual feature extractor for most deep learning based pedestrian attribute recognition methods. Channel attention (CAtt) can adaptively adjust the weight of relevant channel features according to the correlation of input features; it is very helpful for improving feature extraction and attribute recognition performance. However, none of the existing pedestrian attribute recognition methods use a CAtt mechanism. In this paper, the most relevant and salient visual features of pedestrian attributes are extracted and re-adjusted using a CAtt mechanism. The CAtt is seamlessly integrated with ConvLSTM since the CAtt weights for feature re-adjusting are calculated from both visual features and the hidden stats of the ConvLSTM. As far as we know, this is the first time that a CAtt mechanism has been used in pedestrian attribute recognition.
- For multi-label CNN-RNN methods, the prediction sequence of labels (attributes) is important. Most existing methods use a random sequence. In this paper, an optimized prediction sequence is proposed. Considering different area sizes, attributes contain different amounts of information; global attributes (such as gender, age range, etc.) have a larger amount of information, whereas local attributes (such as hair, footwear, etc.) have a smaller amount of information. Attributes with larger amounts of information are easier to recognize accurately. Corresponding to the intuition that easier attributes should be predicted first to help predict more difficult attributes, an optimized prediction sequence from global attributes to local attributes has been put forward to further improve the performance of attribute recognition.
- Extensive experiments are carried out to analyze and verify this method. In-depth comparisons are conducted with seven other state-of-the-art (SOTA) models on two common pedestrian attribute benchmark datasets, PETA [16] and RAP [17]. Compared with these models, the CNN-CAtt-ConvLSTM model proposed in this paper yields superior performance.

## 2. Related Works

Given a person’s image, pedestrian attribute recognition aims to predict a group of attributes to describe the characteristics of the person from a pre-defined attribute list. The task can be handled using different methods, and the methods can be divided into two groups: hand-crafted feature based methods and deep learning based methods.

### 2.1. Pedestrian Attribute Recognition with Hand-Crafted Features

Previously, attribute recognition mainly used manual features [16,18], and separate classifiers were designed for each attribute. Traditional manual features struggle to cope with the complicated and variable surveillance scene and pedestrian appearance by ignoring the relationship between attributes. The correlation between attributes can be used as a constraint to make up for the missing information due to noise in the image. Scholars propose methods based on the graph model [19,20,21], using Markov random field. However, this method needs to calculate the relationship between different attribute pairs, resulting in too many model parameters and a high calculation cost when there are many pedestrian attributes.

### 2.2. Pedestrian Attribute Recognition with Deep Learning

Recently, deep learning has lead to great achievements in automatic feature extraction from multi-layer nonlinear transformations. It is also widely used in pedestrian attribute recognition. In this implementation, image part based methods are often used. Gkioxari et al. [22] and Zhang et al. [23] combined a part based model with CNN to train them with pose normalization for attribute classification. Zhu et al. [24] proposed a multi-label CNN (MLCNN) model that divides pedestrian images into multiple overlapping parts; one model is trained for each part, and the corresponding parts are selected for attribute prediction. Li et al. [17] put forward deep learning based multiple attributes recognition. By combining the whole image and image parts, the whole pedestrian image and three image parts (head shoulder, upper body and lower body) are put into the model for training. Similar to [17], Fabbri et al. [25] took the whole pedestrian image (shoulder, upper and lower body) as inputs, and the scores of different parts are used to predict the attributes. Wang et al. [26] proposed a joint recurrent learning (JRL) model based on CNN and recurrent neural networks (RNN) which transforms a pedestrian image into a region sequence and the attribute set into a sequence list. LSTM was used to predict the attributes in a sequence. Many other pedestrian attribute recognition methods are only based on the entire pedestrian image for modeling. Li et al. [8] and Sudowe et al. [27] proposed deep learning based multiple attributes recognition (DeepMAR) and an attribute convolution network (ACN) respectively, both of which take the whole pedestrian image as the model input and jointly learn the prediction of all attributes. Liu et al. [12] proposed a multi-directional and attention based network (HP-net) that transmits the multi-layer attention feature map of the deep network to different feature layers, integrates the local features of the lower layer and the information of the upper layer, and improves the prediction ability for attributes with a small image size. Li et al. [28] attempted to leverage the physical structure knowledge of people for pedestrian attribute learning. The method first estimates the key points of a given pedestrian image using a pre-trained pose prediction model. Then, it extracts the physical parts according to these key points. The features of the physical parts and the whole image are all extracted and used independently for attribute recognition. The two scores are then fused together to achieve the final attribute recognition. Zhao et al. [29] proposed two models: recurrent convolutional (RC) and recurrent attention (RA) for pedestrian attribute recognition. The RC model is used to explore the correlations between different attribute groups with the convolutional LSTM model, and the RA model takes the advantage of the intra-group spatial locality and inter-group attention correlation to improve the final performance. Liu et al. [30] put forward the joint connectionist temporal classification (CTC) attention model (JCM) for pedestrian attribute recognition, which could predict multiple attribute values of arbitrary length at a time, avoiding the influence of attribute order in the mapping table.

## 3. Proposed Method

### 3.1. Architecture of the Model

**Figure 1:**
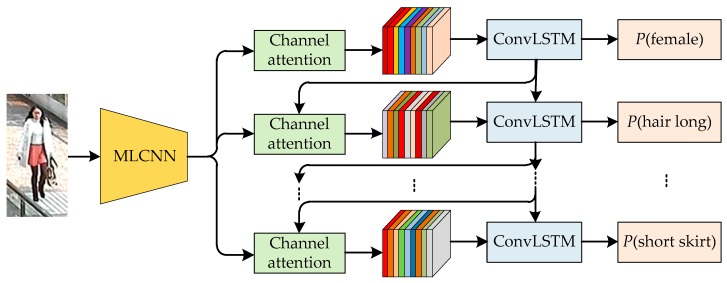
The architecture of proposed convolutional neural networks (CNN), channel attention (CAtt) and convolutional long short-term memory (ConvLSTM) model (CNN-CAtt-ConvLSTM) for pedestrian attribute recognition. MLCNN: multi-label classification CNN.

In this paper, pedestrian attribute recognition is treated as a spatiotemporal, sequential, multi-label classification issue. An attention based neural network (CNN-CAtt-ConvLSTM) model has been designed, and the architecture of the model is shown in Figure 1. It consists of three parts: multi-label classification CNN (MLCNN), a channel attention (CAtt) mechanism and ConvLSTM. MLCNN is used to extract the visual features of the pedestrian image. The mechanism calculates the CAtt weight with self-adaption and adjusts the visual features response to extract the most relevant and salient visual features of the predicted attributes. ConvLSTM further uses the visual features and hidden states for step-by-step pedestrian attribute prediction in an optimized prediction sequence and keeps the context information in the internal memory state to mine the spatiotemporal correlation between attributes.

### 3.2. ConvLSTM in the Model

Pedestrian attributes have strong correlation (i.e., in an image of a person, women and long hair usually appear at the same time, while men and skirts almost never appear together). For the purpose of better exploring the correlation between attributes, this paper considers pedestrian attribute recognition as a sequential task. The CNN-CAtt-ConvLSTM model recognizes pedestrian attributes one by one to fully excavate the correlation between attributes in the process of sequential prediction. As a network that was specifically designed for processing sequence data samples, recurrent neural networks (RNN) not only output each layer to the next layer, but also output a hidden state for the current layer to use when processing the next sample. RNN are good at mining the correlation between samples.

**Figure 2:**
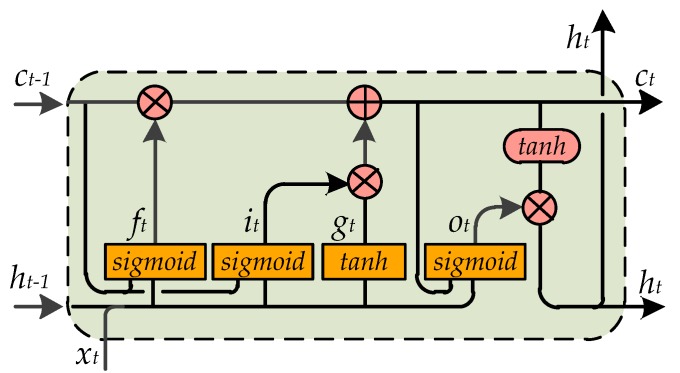
The internal structure of ConvLSTM.

Long short-term memory (LSTM) [31] is an evolutionary version of RNN, which solves the problem of gradient explosion and gradient disappearance in RNN. In this paper, LSTM is used to explore the correlations among the attributes and predict the attributes one by one. When predicting subsequent attributes, LSTM can refer to the hidden state containing historical information. Although LSTM performs well in sequence modeling tasks, the general LSTM ignores spatial information in an image during processing. This is because the general LSTM models the sequence information through the full connection layer and flattens the input image into a one-dimensional vector, which leads to the loss of image spatial information. This is not conducive to the improvement of pedestrian attribute recognition performance. For pedestrian attribute recognition, different image areas have different levels of importance for different attribute prediction (i.e., when predicting hair related attributes, the upper part of the image area is more important, while for footwear related attribute prediction, the lower part of the image area is more important). Retaining relevant spatial information will be very helpful for improving the performance of pedestrian attribute recognition. For the purpose of maintaining the spatial structure of pedestrian attributes, convolutional LSTM (ConvLSTM) [32] is used in our model instead standard LSTM. In ConvLSTM, a convolution operation is used for the conversion of input to state and state to state. It can capture the spatial information of attributes better than standard LSTM, and can therefore better mine the correlation between pedestrian attributes. The formula of ConvLSTM is as follows:

(1) $$\begin{array}{c} i_{t}=sigmoid\left(W_{ix}*x_{t}+W_{ih}*h_{t-1}+W_{ic}⊙c_{t-1}+b_{i}\right)\\ f_{t}=sigmoid\left(W_{fx}*x_{t}+W_{fh}*h_{t-1}+W_{fc}⊙c_{t-1}+b_{f}\right)\\ o_{t}=sigmoid\left(W_{ox}*x_{t}+W_{oh}*h_{t-1}+W_{oc}⊙c_{t}+b_{o}\right)\\ g_{t}=tanh\left(W_{gx}*x_{t}+W_{gh}*h_{t-1}+b_{g}\right)\\ c_{t}=f_{t}⊙c_{t-1}+i_{t}⊙g_{t}\;h_{t}=o_{t}⊙tanh\left(c_{t}\right) \end{array}$$

where ∗ represents the convolution operation and ⊙ represents element multiplication, *sigmoid*(·) represents the logistic sigmoid function and *tanh*(·) represents the hyperbolic tangent function, the subscript *t* represents the *t*-th step of ConvLSTM, $i_{t}$ is input gate, $f_{t}$ is forget gate, $o_{t}$ is output gate, $g_{t}$ is input modulation gate, $x_{t}$ is the input data, $c_{t}$ is the cell state, $h_{t}$ is the hidden state. Here, $x_{t},\;c_{t},\;h_{t},\;i_{t},\;f_{t}\;\mathrm{and}\;o_{t}$ are three-dimensional tensors; the first dimension represents temporal information, and the second and third dimension represent the rows and columns of spatial information. Convolution operation is used here to maintain the spatial information of pedestrian attributes. The core of ConvLSTM is essentially the same as that of general LSTM: the output of the previous layer is taken as the input of the next layer. The main difference lies in that after the convolution operation is added, ConvLSTM can acquire not only the temporal relations but also the spatial relations. This is similar to how the convolution layers in CNN can extract the spatial features in order for the spatiotemporal features to be obtained. In addition, convolutional operations have the effect of spatial attention, because the region which corresponds to the targeted pedestrian attribute normally has a stronger activating response. In our experiments, it is found that because ConvLSTM can focus on the key areas of pedestrian attribute prediction, it can achieve better performance compared to general LSTM. The internal structure of ConvLSTM is shown in Figure 2.

### 3.3. CAtt in the Model

**Figure 3:**
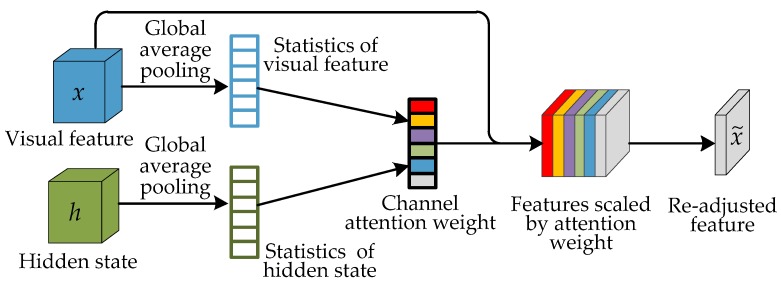
The channel attention mechanism for the proposed CNN-CAtt-ConvLSTM model.

The ConvLSTM described in the previous section has the effect of implicit spatial attention. It can also focus on the key areas related to pedestrian attributes and can effectively improve the performance of pedestrian attribute recognition. Different pedestrian attributes differ not only in the locations of the image, but also in the visual features (i.e., the features of hair and shoes are quite different). The convolution kernel of the CNN has the filtering function of feature (mode) detection. The features of each channel actually represent the weights of the image on different convolution kernels (filters). The role of CAtt is to enhance the feature representation capability of the networks by modeling the dependence of each channel in the feature map, which can be regarded as the process of semantic attribute selection. CAtt first obtains the global information of each channel through global pooling in the channel dimension. Then, it adaptively models the correlation between channels and weights each channel according to the correlation to achieve the goal of feature response and recalibration. In this way, the network can selectively enhance features containing useful information and suppress useless or ineffective features. In this paper, a channel based attention mechanism is designed to use more recognizable and relevant features to perform the task of pedestrian attribute recognition. The spatial attention of ConvLSTM is combined with the channel (feature) attention to further improve attribute recognition performance. The details of the CAtt mechanism are shown in Figure 3. Different from the channel attention in the Squeeze-and-Excitation (SE) network [33], in our CNN-CAtt-ConvLSTM model, the input image features and the hidden states of ConvLSTM are fused for CAtt weight calculation, and CAtt is integrated in the process of ConvLSTM sequential prediction. ConvLSTM will predict pedestrian attribute labels step-by-step and adjust the weight of different feature responses adaptively when predicting different attribute labels.

In order to calculate the attention weight of each feature response channel, this paper uses global average pooling (GAP) to generate the corresponding statistical information of the visual features of each channel as the description of global spatial information based on the channel. In addition, in order to adaptively obtain the CAtt weight based on the previously predicted attribute, the hidden state of ConvLSTM is also considered in the channel based statistics. The calculation formulas of these two categories of statistical information are as follows:

(2) $$m_{c}=g\left(x_{c}\right)=\frac{1}{W\times H}\overset{W}{\underset{i=1}{\sum }}\overset{H}{\underset{j=1}{\sum }}x_{c}\left(i,j\right)$$

(3) $$n_{c}=g\left(h_{t-1,c}\right)=\frac{1}{W\times H}\overset{W}{\underset{i=1}{\sum }}\overset{H}{\underset{j=1}{\sum }}h_{t-1,c}\left(i,j\right)$$

where g(·) represents the GAP function, *c* represents the *c*-th channel, $x_{c}$ and $m_{c}$ represent the visual feature and its statistics, $h_{t-1,c}$ and $n_{c}$ represent the previous hidden state and its statistics, and W and H represent the width and height of the visual feature.

Then, the calculation formula of the channel attention weights is as follows:

(4) $$z_{c}=sigmoid\left(w_{2}ReLU\left(w_{1}\left[m_{c},n_{c}\right]+b_{1}\right)+b_{2}\right)$$

Finally, the input feature is multiplied by the attention weight of each channel to get the re-adjusted feature:

(5) $$\tilde{x}=\overset{C}{\underset{c=1}{\sum }}z_{c}x_{c}$$

### 3.4. Loss Function in the Model

In the surveillance scenario, pedestrian attributes are binary attributes—it is a positive sample if the corresponding attribute appears, and otherwise it is a negative sample. Considering the effectiveness and convenience of the logistic regression function in binary classification, this paper uses a cross entropy loss function based on the sigmoid activation function for multi-attribute classification. In this paper, pedestrian attributes are predicted one by one in an optimized sequence, as detailed in Section 4.5.2. When calculating the loss of each prediction step, firstly, the three-dimensional hidden state of ConvLSTM is flattened into a one-dimensional vector, which is then used for the prediction of pedestrian attributes.

(6) $$p_{j}=sigmoid\left(wh_{j}+b\right)$$

where $p_{j}$ is the predicted output probability of the *j*-th attribute.

The formula of loss function is as follows:

(7) $$Loss=\frac{-1}{N}\overset{M}{\underset{j=1}{\sum }}\overset{N}{\underset{i=1}{\sum }}y_{ij}\log p_{ij}+\left(1-y_{ij}\right)\log\left(1-p_{ij}\right)$$

where *M* represents the number of all pedestrian attribute classes, *N* represents the number of training samples, $y_{ij}$ represents the ground truth (it indicates whether the *j*-th attribute of the *i*-th sample appears; it is equal to 1 if appears, otherwise it is equal to 0), and $p_{ij}$ is the output prediction of the *j*-th attribute of the *i*-th sample.

The loss function formula (Equation (7)) considers all attributes in a unified way and ignores the unbalanced distribution of positive and negative samples in attributes. In the pedestrian attribute datasets (such as PETA and RAP), the positive and negative samples of most attributes are unevenly distributed. As a loss function, Equation (7) will result in samples with a large number contributing most of the loss value, making the gradient descent direction of the neural network continuously tend to the direction in which the attributes with more samples can be predicted correctly, and leads to the accuracy for the attributes with less samples becoming too low. In this paper, the weighted loss function is utilized to resolve the issue, and the following loss function can be obtained through modifying Equation (7):

(8) $$Loss=\frac{-1}{N}\overset{M}{\underset{j=1}{\sum }}\overset{N}{\underset{i=1}{\sum }}w_{1}y_{ij}\log p_{ij}+w_{2}\left(1-y_{ij}\right)\log\left(1-p_{ij}\right)$$

where $w_{1}=\exp\left(1-r_{j}\right)$, $w_{2}=\exp\left(r_{j}\right)$ are the weights, and $r_{j}$ is the positive sample ratio of attribute *j*.

## 4. Experiments and Discussions

### 4.1. Datasets

For effective evaluation, two large public pedestrian attributes datasets, PETA [16] and RAP [17], are used in the experiment. There are a large number of pedestrian images with low resolution, occlusion and clutter in these two datasets, which poses a great challenge to pedestrian attribute recognition.

The PETA dataset is constructed from 10 open pedestrian datasets, including 19,000 pedestrian images in total. Every image has 65 attributes, including 61 binary attributes and four multi-value attributes. The resolution of each image is from 17 × 39 to 169 × 365. Compared with other public datasets, PETA has the following features. (1) Large data volume: the number of samples in the PETA dataset is more than five times that of the Attributed Pedestrians in Surveillance (APiS) dataset and more than 15 times that of the Viewpoint Invariant Pedestrian Recognition (VIPeR) dataset. (2) Data diversity: the PETA dataset selects small-scale datasets in different scenarios and conditions, and the images were taken from various camera angles and various lighting scenes. (3) Innovation of attributes: the PETA dataset has 65 attributes, and 15 of them are valuable attributes used by US officials to identify terrorists.

The RAP dataset is collected from real video surveillance scenes. This dataset not only marks pedestrian attributes, but also environmental and situational factors. There are 41,585 pedestrian images in the RAP dataset, where 72 attributes are marked for each pedestrian, including 69 binary attributes and three multi-value attributes, including shooting angle, occlusion, body part information and some attributes suggested by the police. It is the biggest pedestrian attribute dataset currently available. The dataset highlights environmental and situational factors. The video comes from 26 cameras with a resolution of 1280 × 720 and a frame rate of 15, and the video data are captured for three consecutive months. Different from other datasets (such as PETA) which collect data from different data sources, the data in RAP are all from real video surveillance scenes, which is closer to real application scenes.

### 4.2. Evaluation Metrics

In this paper, two methods and four criteria are used to evaluate the performance of pedestrian attribute recognition.

Class-based evaluation: for every attribute class, the classification accuracy of its positive and negative samples are calculated separately. Its average value is used as the average accuracy of the attribute class, and then the mean accuracy (mA) of all attribute classes as an evaluation criterion is calculated [21].

Instance-based evaluation: the above class-based evaluation method handles every attribute independently, ignoring the correlation between attributes. Therefore, this paper also uses the instance-based evaluation method to measure attribute prediction precision and the recall rate of each instance (image). Unlike mA, which assumes that attributes are independent of each other, the instance-based evaluation method also considers the correlation between attributes. In this paper, according to the labeled ground truth, the precision and recall rate of each test image are calculated, and then the precision and recall rate of all test images are calculated to get the mean precision (mP) and the mean recall rate (mR). In addition, the integrated value of mP and mR with balancing factor equals to 1 (F1) is calculated [17], and the four indexes of mA, mP, mR and F1 are used for comprehensive evaluation.

### 4.3. Implementation Details

Tensorflow is used in the implementation of the model, and Keras is used as the high-level neural network API on top of Tensorflow to speed up the implementation and experimentation. The detailed software environment is as follows: Ubuntu 18.04, Python 3.6.7, CUDA 10.0, CUDNN 7.5, Tensorflow-GPU 1.13.1, Keras 2.2.4.

MLCNN uses the multi-label version of Inception-v3 [34]. We use the Inception-v3 model provided by Keras applications with weights pre-trained on ImageNet. The outline of the network structure of Inception-v3 can be found in Table 1 of reference [34]. The output layer of Inception-v3 is replaced with M neurons (M represents the number of all pedestrian attribute classes) to transform Inception-v3 into a multi-label classification framework (MLCNN) where multi-label sigmoid cross-entropy loss function is used to replace the softmax function. The input is RGB three-channel images resized to 299 × 299. For the RAP dataset, the image is only resized using the bilinear interpolation method. For the PETA dataset, as there are relatively few training images, the data is augmented using random left-right flips and image color jitter, including adjustments to saturation, brightness and contrast.

In order to accelerate convergence, a two-phase training method is adopted during implementation. During the first phase, the main task is to train the MLCNN. The multi-label sigmoid activation function and cross entropy loss function are used to fine tune the MLCNN, which was pre-trained based on ImageNet. In the second phase, MLCNN’s full connection layer is removed and other parameters are fixed. Then, based on the features extracted by MLCNN, ConvLSTM and CAtt are trained from scratch. Among the 19,000 pedestrian images from the PETA dataset, we randomly divided the whole dataset into three non-overlapping partitions: 9500 for model training, 1900 for verification, and 7600 for model evaluation. We evaluate the same 35 binary attributes as [16]. As for RAP dataset, we adopted the same data split as in [17]: 33,268 images for training and the remaining 8317 for testing. We evaluate the same 51 binary attributes as [17] for a fair comparison. For both datasets, we converted multi-valued attributes into binary attributes as in [17,21]. The two datasets are trained and tested separately since cross dataset (domain) learning is another big challenge of pedestrian attribute recognition tasks and is out of the scope of this paper.

The batch size is set to 32 for both phases, and epoch is 100 for the first phase and 80 for the second phase. The Stochastic Gradient Descent (SGD) with momentum optimizer is used in both phases, and for the momentum value, the first phase is 0.5 and the second phase is 0.99. In order to avoid over fitting, dropout and Ridge Regression (L2) regularization are used in both phases. The dropout rate is set to 50% and L2 regularization weight is set to 0.005. The initial learning rate is 0.01, which decays ten times after loss stabilization.

### 4.4. Comparison with Other Methods

This section compares our method with seven other state-of-the-art (SOTA) methods based on deep learning, including three methods based on CNN and four methods based on the CNN-RNN joint model. (1) Sudowe et al. [27] proposed ACN (joint training of all attributes, setting a loss function for each attribute separately, and weighted average of all loss functions as the total loss value). (2) Li et al. [8] proposed DeepMAR to jointly train all attributes and capture the correlation between attributes. (3) HP-net [12] is a deep model based on the attention mechanism, which uses the Inconcept-v2 network structure to map multi-layer attention maps to multi-layer network blocks, so that the model can integrate global and local information. (4) Li et al. [15] put forward a sequential prediction solution which uses the image context information based contextual CNN-RNN (CTX) model, encodes scene context information, explores the relationship between pedestrians, and models multiple pedestrians in the same image. (5) Liu et al. [35] proposed a semantic regularized (SR) CNN-RNN model to deal with multi-label image classification problems and select more robust image features. (6) Wang et al. [26] proposed JRL, and used a recurrent neural network to explore the relationship between all attributes of the same pedestrian and different pedestrians. (7) Zhao et al. [29] proposed a model combining recurrent learning with attention (RA) that highlights the spatial location of feature maps and excavates the attention correlation between different attribute groups for the purpose of obtaining more accurate attention.

The experimental results of this method compared with other SOTA methods are shown in Table 1. The following conclusions can be reached according to the analysis: (1) our method achieved the best result on mA, mR and F1 indexes on both the PETA and RAP datasets, and a similar performance for mP indexes as DeepMAR, which achieves the best performance in mP. (2) JRL and RA also achieve relatively good performance on both datasets among the seven competitors. Interestingly, JRL is based on LSTM with attention, RA is based on ConvLSTM with attention, and both models are similar to our model (which achieves better performance). This phenomenon proved the validity of our method.

In summary, the experimental results clearly show the advantages of our model for pedestrian attribute recognition. This is primarily because our model can not only effectively pay attention to the most relevant attribute features, but also maintain the spatial information of visual features for different attribute predictions, which is conducive to fully mining attribute correlations and improving the performance of pedestrian attribute recognition.

### 4.5. Further Analysis and Discussions

#### 4.5.1. The Effect of ConvLSTM and CAtt

For verifying the effectiveness of ConvLSTM and CAtt in the model, our method is compared with the following methods: multi-label CNN (MLCNN), CNN-LSTM, CNN-SAtt-LSTM which has spatial attention (SAtt), CNN-ConvLSTM with no attention, and CNN-SAtt-ConvLSTM with spatial attention. For the purpose of making a fair comparison, the CNN of all methods is based on Inperception-v3.

**Figure 4:**
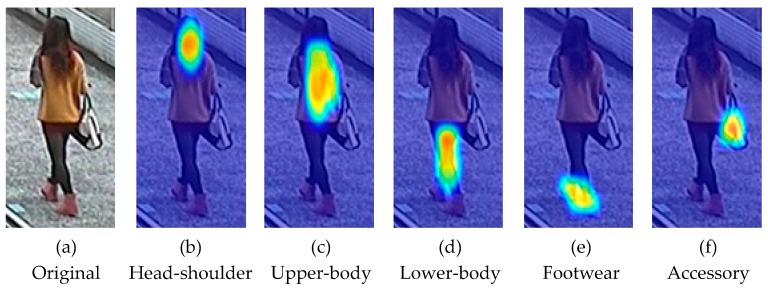
The attention heat map of the CNN-CAtt-ConvLSTM model when predicting pedestrian attributes in different regions. (**a**) is the original pedestrian image, (**b**–**f**) are the attention heat maps of the regions: head-shoulder, upper-body, lower-body, footwear and accessory.

The experimental results are shown in Table 2. It can be seen that our model achieves the best performance. When CNN-LSTM does not have the attention mechanism, its performance declines substantially, which shows the importance of different areas to different attributes in pedestrian attribute recognition and the effectiveness of the attention mechanism. The results of CNN-ConvLSTM and CNN-SAtt-LSTM are similar, which shows the effectiveness of ConvLSTM in the extraction of spatial information from related areas. Figure 4 shows an example of the attention heat maps of our model when predicting different pedestrian attributes; it shows that ConvLSTM has a high activation response to the image area corresponding to the attribute when predicting the different pedestrian attributes, indicating that the convolution operation of ConvLSTM actually has the effect of implicit spatial attention. The result of CNN-SAtt-ConvLSTM is slightly better than that of CNN-ConvLSTM which has no attention mechanism, but is much worse than that of our model which contains ConvLSTM and CAtt. The main reason for this is that the SAtt mechanism overlaps with the implicit spatial attention ability of ConvLSTM, which has little improvement on the performance of pedestrian attribute recognition. The channel attention mechanism can re-adjust the weights based on the feature correlation and salience, combined with the spatial correlation ability of ConvLSTM, which results in an effective improvement for the performance of pedestrian attribute recognition. Overall, our proposed method obtained better performance than other methods, which proved the superiority of our method.

#### 4.5.2. The Effect of Optimized Prediction Sequence

In order to fully mine the correlation of pedestrian attributes to improve performance, this paper takes pedestrian attribute recognition as a spatiotemporal sequential problem and uses ConvLSTM to predict attributes one by one. Thus, the prediction result of the previous attributes will have an impact on the prediction of the subsequent attributes, and the correct prediction of the previous attributes will be conducive to the prediction of the subsequent attributes. Conversely, if the prediction of the previous attributes is wrong, it will mislead the prediction of the subsequent attributes. As the prediction sequence is a key consideration that influences the performance of pedestrian attribute recognition, it is important to carefully choose the proper prediction sequence. In general, the prediction accuracy of the attributes with a larger area is higher than that of the attributes with a smaller area because a larger area usually has a larger amount of information. Therefore, this paper proposes an optimized prediction sequence which analyzes the size of the area for each attribute in the datasets in advance, and then according to the attribute area and combined with the different importance of each attribute in the video surveillance footage, determines the sequence of each attribute prediction.

In this paper, pedestrian attributes are first divided into two groups: one is global attributes, (i.e., gender, age range and body shape) and the other is local attributes (i.e., hair style, upper body color and hand-held items). Global attributes usually need to be predicted from all areas of a pedestrian image, whereas local attributes can be predicted from local areas of a pedestrian image. In this paper, global attributes are predicted first and then local attributes are predicted. Global attributes can usually be predicted without relying on other attributes. For example, even if many other attributes are unclear, gender and age are easier to identify. Therefore, global attributes should be predicted at the beginning of the sequence to avoid being misled by wrong local attributes. Predicting global attributes first is very helpful to predict other correlated local attributes. For example, if a pedestrian’s gender is recognized as a female, she is more likely to have long hair. Therefore, when the hair length attribute is predicted after the gender attribute is recognized, the probability of the attribute being correctly recognized is higher. By combining the size of the attribute area and its importance in surveillance, the attribute prediction sequence can finally be determined. Taking the PETA dataset as an example, the prediction sequence can be: gender > age range > hair length > upper body style > lower body style > footwear style > hair color > upper body color > lower body color > footwear color > backpack > handbag > hat > headphones, etc.

The experimental result of the optimized prediction sequence with a comparison to that of a random sequence is shown in Table 3. The experimental results listed in Table 3 show that the performance of the optimized sequence is better than that of the random sequence.

## 5. Conclusions

In this paper, a novel attention based neural network model (CNN-CAtt-ConvLSTM) is proposed to fully mine the semantic correlation and spatial information of pedestrian attributes to improve the performance of pedestrian attribute recognition. In this model, MLCNN and CAtt are used to extract the most relevant visual features of the predicted pedestrian attributes. The CAtt mechanism is further combined with ConvLSTM to mine the correlation between attributes and the spatial information of the visual features of attributes. Pedestrian attributes are predicted one by one based on the prediction sequence optimized by the area size and the importance of attributes. Experimental results indicate that our method obtains better performance than most other existing pedestrian attribute recognition methods, which confirms the effectiveness and performance advantage of this method. In future work, instead of just using the implicit spatial attention capability provided by ConvLSTM, we will try to modify the internal door structure of ConvLSTM and explore the different combinations of attention mechanism and ConvLSTM (i.e., explicitly put the attention mechanism in different places of ConvLSTM so as to more effectively mine the semantic and spatial correlation of pedestrian attributes to further improve the performance of pedestrian attribute recognition). Additionally, the optimized prediction sequence in this work is fixed and static based on the prior analysis on the dataset; it is not flexible enough for new datasets. In future work, we will try to make the prediction sequence automatically decided and dynamic based on the correlation between attributes. The next attribute sequence will be automatically adjusted according to the recognition result of the previous attribute, and the performance of pedestrian attribute recognition will be further improved.

## Figures and Tables

**Table 1:** Comparison with state-of-the-art (SOTA) methods on the PETA and RAP datasets.

	Metric	PETA				RAP			
Method		mA	mP	mR	F1	mA	mP	mR	F1
ACN [27]		81.15	84.06	81.26	82.64	69.66	80.12	72.26	75.98
DeepMAR [8]		81.50	89.70	81.90	85.68	76.10	82.20	74.80	78.30
HP-net [12]		81.77	84.92	83.24	84.07	76.12	77.33	78.79	78.05
CTX [15]		80.13	79.68	80.24	79.68	70.13	71.03	71.20	70.23
SR [35]		82.83	82.54	82.76	82.65	74.21	75.11	76.52	75.83
JRL [26]		85.67	86.03	85.34	85.42	77.81	78.11	78.98	78.58
RA [29]		86.11	84.69	88.51	86.56	81.16	79.45	79.23	79.34
Ours		88.56	88.32	89.62	88.97	83.72	81.85	79.96	80.89

**Table 2:** Experimental result on the effect of ConvLSTM and CAtt.

	Metric	PETA				RAP			
Method		mA	mP	mR	F1	mA	mP	mR	F1
MLCNN		79.86	81.73	79.92	80.81	68.22	72.46	71.34	71.90
CNN-LSTM		81.63	83.25	82.54	82.89	74.63	75.97	76.62	76.29
CNN-SAtt-LSTM		85.13	85.75	84.95	85.35	77.49	77.85	78.32	78.08
CNN-ConvLSTM		85.92	85.21	86.12	85.66	79.35	78.73	78.65	78.69
CNN-SAtt-ConvLSTM		86.08	85.34	86.22	85.78	79.48	78.83	78.77	78.80
CNN-CAtt-ConvLSTM (Ours)		88.56	88.32	89.62	88.97	83.72	81.85	79.96	80.89

**Table 3:** Experimental results on the effect of the optimized prediction sequence.

	Metric	PETA				RAP			
Method		mA	mP	mR	F1	mA	mP	mR	F1
Random sequence		88.01	87.81	89.13	88.47	83.13	81.32	79.46	8038
Optimized sequence		88.56	88.32	89.62	88.97	83.72	81.85	79.96	80.89
